# The Ellipsoid Factor for Quantification of Rods, Plates, and Intermediate Forms in 3D Geometries

**DOI:** 10.3389/fendo.2015.00015

**Published:** 2015-02-16

**Authors:** Michael Doube

**Affiliations:** ^1^Department of Comparative Biomedical Sciences, The Royal Veterinary College, London, UK

**Keywords:** maximally inscribed ellipsoid, optimization, segmentation, plate, rod, Tb.EF

## Abstract

The ellipsoid factor (EF) is a method for the local determination of the rod- or plate-like nature of porous or spongy continua. EF at a point within a 3D structure is defined as the difference in axis ratios of the greatest ellipsoid that fits inside the structure and that contains the point of interest, and ranges from −1 for strongly oblate (discus-shaped) ellipsoids, to +1 for strongly prolate (javelin-shaped) ellipsoids. For an ellipsoid with axes *a* ≤ *b* ≤ *c*, EF = *a*/*b* − *b*/*c*. Here, EF is demonstrated in a Java plugin, “Ellipsoid Factor” for ImageJ, distributed in the BoneJ plugin collection. Ellipsoid Factor utilizes an ellipsoid optimization algorithm, which assumes that maximal ellipsoids are centered on the medial axis, then dilates, rotates, and translates slightly each ellipsoid until it cannot increase in volume any further. EF successfully identifies rods, plates, and intermediate structures within trabecular bone, and summarizes the distribution of geometries with an overall EF mean and SD, EF histogram, and Flinn diagram displaying *a*/*b* versus *b*/*c*. EF is released to the community for testing, use, and improvement.

## Introduction

The plate- and rod-like shapes observed within trabecular bone may be mechanically important yet represent a challenge to quantification because they are difficult to define and identify in a meaningful way (1–9). The *de facto* standard is the “structure model index” (SMI) which classifies surfaces based on their change in surface area after an infinitesimal dilation, and works well for perfect spheres and cylinders, and other curves of a purely convex nature (9). However, real bone contains a large proportion of concave curvature, and saddle curvature (concave in one direction and convex in the other), which vary as a function of bone volume fraction (BV/TV). In these cases, SMI does not perform well and the final reported figure contains “contamination” from concave portions of the surface. BoneJ (10) implements SMI and uniquely reports the negative contribution of concave surfaces to the SMI sum.

A number of improvements to SMI have been proposed, including Stauber and Müller’s volumetric spatial decomposition and Liu et al.’s individual trabecular separation approaches (1, 4). The emphasis of these algorithms is on splitting the structure into discrete elements, then reporting the properties of each element and summing their contribution to the whole. However, except in the most extreme cases, trabecular bone operates as a continuum (a “cellular solid” (11)) rather than as a set of discrete nodes and struts and so the discretization of bony continua into “plates” and “rods” may be artificial as a general solution. In particular, it seems inappropriate when the topology is complex, containing oblique branching and perforation, and when BV/TV rises so that rods and plates are no longer clearly discernible.

## Development

**Figure 1:**
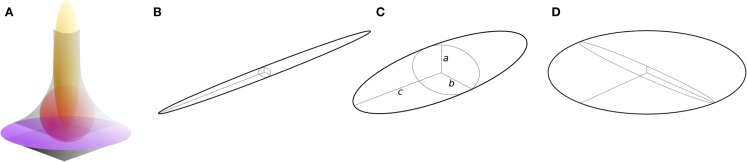
**Ellipsoids of differing proportions**. **(A)** In curved geometries (gray), prolate [javelin-shaped, yellow, **(B)**] ellipsoids maximally fit rod-like regions, intermediate ellipsoids [red, **(C)**] maximally fit junction regions, and oblate [discus-shaped, purple, **(D)**] ellipsoids maximally fit plate-like regions. Ellipsoids have three semi-axes (radii), *a*, *b*, and *c* **(C)**. Prolate ellipsoids **(B)** have one long radius and two short radii such that *a* ≤ *b* ≪ *c*, while oblate ellipsoids **(D)** have two long radii and one short radius such that *a* ≪ *b* ≤ *c*. Intermediate ellipsoids **(C)** have more moderately differing radii, *a* ≤ *b* ≤ *c*. The ellipsoid factor (EF) of an ellipsoid is calculated as EF = *a*/*b* − *b*/*c*.

Here, I present an approach for the local classification of three-dimensional continua such as trabecular bone based on fitting maximal inscribed ellipsoids. It is intended to overcome limitations of previous concepts and to provide a general solution that makes few assumptions, and that treats bone as a continuum. It is an intuitive extension of the approach used to measure trabecular thickness (Tb.Th), in which the thickness at a point is the diameter of the largest sphere that contains the point and that fits inside the structure (12). The ellipsoid factor (EF) is similarly defined as the difference in axis ratios of the largest ellipsoid, which contains the point and which fits inside the structure. For an ellipsoid (Figure 1C) with three semi-axis lengths (“radii”) a, b, and c:

(1) $$a\leq b\leq c,\;\mathrm{EF}=\frac{a}{b}-\frac{b}{c}$$

It can be seen from this definition that both *a*/*b* and *b*/*c* have minimum and maximum values of 0 and 1, and so EF can take values from −1 to +1. EF of −1 means that *a*/*b* → 0 and *b*/*c* → 1: this occurs in extremely oblate (discus-shaped) ellipsoids, which have one short axis and two long axes (Figure 1D). EF of +1 means that *a*/*b* → 1 and *b*/*c* → 0: this occurs in extremely prolate (javelin-shaped) ellipsoids, which have one long axis and two short axes (Figure 1B). EF of 0 means that *a*/*b* = *b*/*c*, which is an intermediate state between “discus” and “javelin” – spheres satisfy EF = 0 (a:b:c = 1:1:1) along with ellipsoids with axis ratios 1:2:4, 1:3:9, or more generally, a:*q*a:*q*^2^a. A strongly prolate ellipsoid (javelin) would maximally fit a very rod-like feature, while a strongly oblate ellipsoid (discus) would maximally fit a very plate-like feature (Figure 1A). The EF at a particular point in the structure is determined by a competition between the overlapping ellipsoids that contain the point. To maintain consistency with the original definition of Local Thickness, here the ellipsoid competition is won by the ellipsoid of greatest volume.

## Implementation

A proof-of-concept implementation has been developed in Java as an ImageJ plugin, and is available, including source code, as part of BoneJ [v1.4.0 and later (10)^1^]. All code changes are publicly available from GitHub^2^, with developments pushed to the ellipsoid-factor branch, prior to merging with the master branch for general release. Anyone may make improvements to the code, and developers are strongly encouraged to share their changes with the parent project.

Input data are required to be a three-dimensional binary stack. A topology-preserving medial axis thinning is performed with ImageJ’s Skeletonize3D plugin (13, 14) and the resulting skeleton points are stored as seed points. For each seed point, a small spherical ellipsoid is instantiated (see BoneJ’s Ellipsoid class). A fixed number (default, *n* = 100) of points are drawn on the ellipsoid’s surface and the value in the input image at each surface point’s coordinate is tested. The ellipsoid is dilated equally along its three axes until at least one surface point lies on a background pixel. When an ellipsoid surface point lies on a background pixel, it is designated as a “contact point.” The ellipsoid is then rotated so that one axis aligns with the mean unit vector pointing to the contact point(s), and contracted to lie completely within the foreground. The other two axes are then dilated equally until the number of contact points is at least 1, and the ellipsoid rotated so that a second axis is aligned with the mean vector of contact points.

An iterative cycle of dilation, rotation/translation, and contraction follows. During each iteration, the ellipsoid’s volume is determined and if it is the maximal volume found so far, the ellipsoid is stored and used for subsequent iterations. Iteration continues until no further increase in volume has been achieved after a user-set number of iterations. Within each iteration, each axis is dilated individually, and the ellipsoid is rotated by a small random rotation [wiggle()], translated [bump()], or turned [turn()] before being contracted until fully fitting within the foreground. Total translation is limited in magnitude so as to prevent ellipsoids drifting far from their seed point, but allowing them to overcome pixelation artifacts relating to discretized 3D space. Translation direction is the mean of the unit vectors from contact points to the center. The turn direction is the torque of unit vector surface normals pushing on the contact points. The general effect of turn() and bump() is for the object boundary to “push back” upon the growing ellipsoid, so that it grows into unfilled space where it is not contacting the boundary. The wiggle() method is included to allow the ellipsoid to overcome ridges and to find growth pathways that might not be found by a strictly analytical approach.

Coordinates outside the image bounds are considered background. Input pixels on the image boundaries tend to seed ellipsoids, which can grow without limit into the out-of-bounds volume. To prevent uncontrolled growth causing a computational halting problem and to avoid results being overly influenced by out-of-bounds space, ellipsoids are culled if they are >50% outside the image bounds; if their volume exceeds the image stack volume; or if their volume has not stabilized before a user-set, large number of iterations.

Following iterative maximal inscribed ellipsoid fitting, an array of all the optimized ellipsoids is formed and sorted in descending order of volume. For each foreground pixel coordinate of the input image, the ellipsoid array is iterated until the first ellipsoid that contains the pixel is found. Because the array is sorted on volume, the first ellipsoid found to contain the point is guaranteed to be the largest. The array index of this ellipsoid is stored in the location of the foreground pixel, forming a 3D map of maximal ellipsoid array indices. Background pixels are set to a large negative number and unmatched foreground pixels for which there is no containing ellipsoid are given the index −1. Further analysis is performed using the ellipsoid array cross-referenced from the 3D map of array indices.

Ellipsoid fitting is substantially more complicated than sphere fitting, due to the additional degrees of freedom. Whereas a sphere is defined by its center and radius alone, an ellipsoid is defined by its center *p*_c_ = (*x*_c_, *y*_c_, *z*_c_), a 3 × 3 eigenvalue matrix whose diagonal values λ_1_, λ_2_, and λ_3_, relate to the three semi-axis lengths *r*_a_, *r*_b_, *r*_c_ as:

(2) $$r=\frac{1}{\sqrt{\lambda }}$$

and a 3 × 3 eigenvector rotation matrix. BoneJ’s Ellipsoid class uses the matrix definition of an ellipsoid, rather than the quadratic equation form:

(3) ax2+by2+cz2+2 dxy+2 fxz+2 gyz+2 hx+2 jy+2 kz=1

because the matrix form makes translation and rotation transformations trivial to implement and fast to execute. The matrix form also allows fast determination of whether a point lies inside or on an ellipsoid by satisfying the inequality:

(4) $${\left(X-X_{0}\right)}^{T}H\left(X-X_{0}\right)\leq 1$$

where *H* is the product of the eigenvalue and eigenvector matrices, *X* is the test point, and *X*_0_ is the center. To simplify calculation and reduce the number of ellipsoids to optimize, this implementation enforces, like Local Thickness does with spheres (15), that the maximal ellipsoids are centered on the medial axis.

## Results and Use

**Figure 2:**
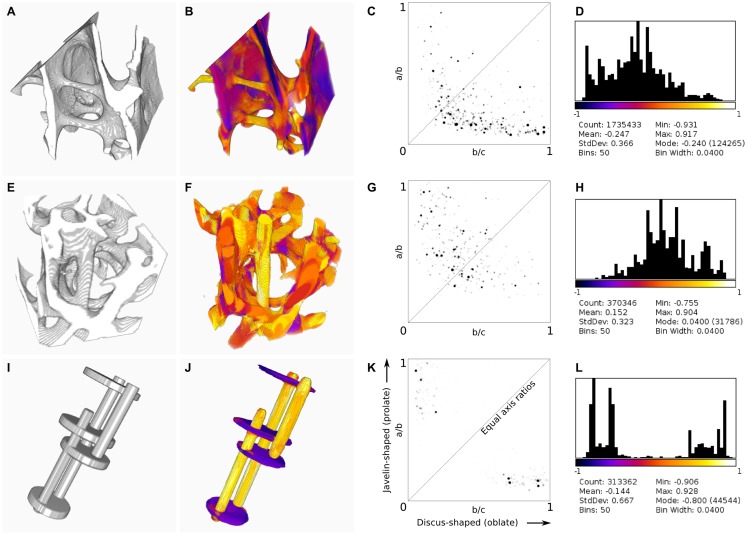
**Results of BoneJ’s ellipsoid factor implementation, run on X-ray microtomographic images of trabecular bone from the femora of emu [*Dromaius novaehollandiae*, (A–D)] and lesser dwarf shrew [*Suncus varilla* (E–H)], and on a synthetic image of rods and plates (I–L)**. Input geometry **(A,E,I)** was processed using default settings, except that all skeleton points were used (default is to use only every 50th point). 3D color map images **(B,F,J)** indicate EF > 0 in orange–yellow and EF < 0 in purple–blue [look-up table is the same as in **(D,H,L)**]. Note the labeling of rods in orange–yellow and plates in purple–blue. Flinn diagrams **(C,G,K)** demonstrate the distribution of axis ratios toward the top left for rod-dominated structures **(G)** and the bottom right for plate-dominated structures **(C)**. **(K)** shows discrete clusters of peaks relating to the rods and plates in the synthetic image. The diagonal indicates the line where *a*/*b* = *b*/*c*; ellipsoids are spherical at the top right corner. Histograms and summary statistics of EF **(D,H,L)** display a shift to the left for plate-dominated structures **(D)**, to the right for rod-dominated structures **(H)** and a bimodal distribution for structures with few intermediate ellipsoids **(L)**.

The Ellipsoid Factor plugin was tested on a Dell T7600 workstation (Dell Products, Bracknell, Berkshire, UK) with 12 CPU cores using three binary stack images, which are available for download at http://bonej.org/ef (Table 1, Figures 2A,E,I). Two images are X-ray microtomography scans from a previous study (16) that provide a variety of natural geometry. Specifically, trabecular bone from an emu (*Dromaius novaehollandiae*) was selected because it contains large plates separated by well-defined rods (Figure 2A), and trabecular bone from a shrew (*Suncus varilla*) was selected to provide a rod-dominated volume (Figure 2E). A further synthetic image was constructed by filling hand-drawn ROIs to produce a 3D volume with a small number of intersecting rods and plates (Figure 2I). The absolute pixel spacing of these images is irrelevant to EF because being derived from ratios it is a unitless measure; however, the ratio of pixel spacing to feature size is critically important: EF is likely to function decreasingly well as pixel spacing approaches feature size. Mean feature size is reported in Table 1 as Tb.Th and varies from 14.1 to 16.1 pixels.

**Table 1:** **Comparison of ellipsoid factor to SMI, BV/TV, and Tb.Th**.

Image	Size (px)	EF	SMI	SMI+	SMI−	BV/TV	Tb.Th (px)	*t*_EF_ (s)	*t*_Tb.Th_ (s)
Emu (Figure 2A)	239 × 242 × 201	−0.247	1.140	1.610	0.470	0.157	16.1	540	3.40
Shrew (Figure 2E)	114 × 114 × 115	0.152	2.076	2.472	0.396	0.293	14.8	21.3	1.24
Synthetic (Figure 2I)	128 × 128 × 256	−0.144	2.004	2.272	0.268	0.098	14.1	7.72	0.94

*Results of running BoneJ’s prototype ellipsoid factor implementation on a 12-core Dell T7600 workstation on three test images. Note the inconsistent relationship between EF and SMI, and the strong negative component to SMI (SMI−), which is nearly 30% of the positive component (SMI+) in the emu image, despite a relatively low volume fraction (BV/TV). The features in these example images are well sampled with a mean thickness of 14.1–16.1 pixels. Processing time increases exponentially for EF (*t*_EF_), compared to Tb.Th (*t*_Tb.Th_), which indicates that improved optimization strategies are required for future EF implementations. All images are available to download from http://bonej.org/ef*.

Ellipsoid fitting successfully identifies rod- and plate-like regions within trabecular bone, with over 90% of pixels classified by at least one ellipsoid (Figure 2). Ridge features, which are neither rod nor plate in the intuitive sense, and which would challenge trabecular separation techniques, are successfully identified. Segmentation of the structure based on EF, or on the individual *a*/*b* and *b*/*c* ratios, is possible, and reveals rod-like features within plates and plate-like regions within rods. Junctions between trabecular elements are identified by intermediate ellipsoids.

Ellipsoid factor can be used as a global measure, like SMI, summarizing the geometry of the whole by calculating the mean pixel value of the EF image. EF forms a much more natural variable than SMI to take the mean of because it varies between −1 and +1 and, in concert with the axis ratio distribution data, the mean ellipsoid can be calculated and displayed directly, unlike SMI, which varies non-linearly from 0 to 4 and for which the resulting mean geometry is difficult to visualize meaningfully. It must be noted that EF, as a simple summary variable, cannot by itself distinguish between a structure formed of equal amounts of rod-like (*a*/*b* → 1; *b*/*c* → 0, EF → 1) and plate-like (*a*/*b* → 0; *b*/*c* → 1, EF → −1) ellipsoids, and a structure composed of intermediate ellipsoids (where *a*/*b* = *b*/*c*), because in both conditions the volume-weighted EF of the overall structure tends toward 0. For this reason, an EF histogram should be constructed and interpreted alongside the Flinn diagram, which displays how much of the volume of the structure is described by ellipsoids of particular axis ratios.

The Flinn diagram is more commonly used to model strain and constant volume deformation in geological structures (17, 18), with axis ratios formed by the eigenvalues of the strain tensor. It is a convenient and intuitive method for displaying and analyzing ellipsoid geometry. Here, the Flinn diagram plots the ratios of ellipsoid semi-axis lengths (themselves measured in real spatial units), and for consistency places prolate ellipsoids to the top left and oblate ellipsoids to the lower right.

## Discussion

Ellipsoid fitting is much more challenging than sphere fitting, because a sphere is defined by a center and radius, whereas an ellipsoid is defined by a center, three semi-axes, and a rotation. These additional degrees of freedom mean that searching for optimally fitting maximally inscribed ellipsoids is non-trivial in comparison to searching for maximally inscribed spheres, which itself is a computationally intensive task. Here, complexity is reduced by assuming that the maximal ellipsoids are centered on the medial axis, as maximal inscribed spheres are, and so ellipsoids are seeded only from the medial axis and not from every point in the structure.

The algorithm described here is a proof of concept that runs sufficiently well to demonstrate the utility of EF in quantifying the rod- and plate-like nature of trabecular bone geometry. However, it must be noted that in its current form it contains some important limitations. The first is speed: the iterative method is relatively slow, requiring tens to hundreds of milliseconds on contemporary hardware to fit each ellipsoid. This is mitigated to some degree by operating in parallel, so that each ellipsoid is optimized independently in a separate thread of the CPU. Large datasets in particular benefit from an approximately linear speedup by increased number of CPU cores. Further speed improvements might be possible by offloading some of the calculations to the GPU, but this may require that the whole image dataset is stored in graphics RAM.

The simple point-probe method used here, where each ellipsoid is tested based on a fixed number of approximately equally spaced points on its surface, has the side-effect of potentially under- or oversampling features in the geometry. This can lead to the optimization ignoring small features such as intratrabecular osteonal canals due to too widely spaced point-probes, or to wasting CPU and memory access cycles due to unnecessarily dense point-probes sampling the same pixel multiple times. An improvement may be to allow the point-probe number and position to vary as a function of ellipsoid size and geometry, so that the inter-point spacing remains similar during ellipsoid dilation and fitting. It may be noted that at the time of its invention, Hildebrand and Rüegsegger’s sphere-fitting implementation took around 15 min to process a 286^3^ pixel volume on an advanced (for the day) workstation (12). Improved algorithms and hardware mean that a similar Tb.Th measurement job today requires only a few seconds to complete (Table 1). I expect that similar improvements in processing speed await EF, should it be found to be useful to the image analysis community.

This implementation, in its current state, does not guarantee complete filling of the input geometry with ellipsoids, unlike the Local Thickness algorithm implemented by Dougherty and Kunzelmann (15). The degree of filling is reported in the log and can be 90% or greater, particularly if all the skeleton points are used to seed ellipsoids and if few or none of the input pixels lie on the image boundaries. An improvement may be to use a seeding strategy other than the current medial axis approach, such as a 3D distance ridge, so that ellipsoids are distributed more evenly throughout the geometry. However, there is still no guarantee that an evenly seeded spherical set of ellipsoids do not optimize away from small surface details due to the optimization strategy, which keeps only the largest discovered ellipsoid centered on the seed point. These residual unclassified pixels might be dealt with by filling them with spheres and giving them an EF of 0, by attaching them to the nearest ellipsoid, or by adding a strategy to the implementation, which aggressively attempts to classify all input pixels by further ellipsoid seeding and fitting.

Finally, the 3D map of EF values is not as smooth as might be expected, which is esthetically disturbing but is an analytically correct result based on the EF definition in which the largest ellipsoid containing a pixel “wins” that pixel. A more balanced and potentially less biased approach might be to allow the nearest ellipsoid to win, or to calculate a distance- or volume-weighted mean at each pixel.

In conclusion, EF is a useful new method for the measurement and segmentation of complicated porous continua such as trabecular bone. It can give a summary of the rod- and plate-like nature of a 3D structure and can identify the dominant geometry at each point within the structure. I suggest the use of the abbreviation Tb.EF to maintain consistency with the standard bone nomenclature (19), when trabecular bone is the input geometry. The current implementation in BoneJ is a working proof-of-concept which the community is encouraged to test and comment on, and upon which improvements in pixel labeling efficiency and computational optimization will be made.

## Conflict of Interest Statement

The author declares that the research was conducted in the absence of any commercial or financial relationships that could be construed as a potential conflict of interest.

## Footnotes

^1^http://bonej.org/ef

^2^http://github.com/mdoube/BoneJ/
